# Analysis of bacteriological pollution and the detection of antibiotic resistance genes of prevailing bacteria emanating from pig farm seepage

**DOI:** 10.1002/mbo3.737

**Published:** 2018-11-09

**Authors:** Dikonketso Shirley‐may Matjuda, Olayinka Ayobami Aiyegoro

**Affiliations:** ^1^ Gastro intestinal Microbiology and Biotechnology Agricultural Research Council‐ Animal Production Irene South Africa

**Keywords:** antibiotics, bacteria, pig farm, pollution, resistance gene, Seepage

## Abstract

Management and disposal of pig farm seepage constitute a serious environmental challenge, and seepage discharge from agricultural waste‐water is considered to be one of the greatest contributors of organic substances, bacterial pathogens, and antibiotic resistance genes into the environment. The objectives of this study were to assess the level of bacteriological pollution and to identify the resident antibiotic‐resistant genes of culturable bacteria from a studied pig farm seepage. Enumeration of the viable bacterial cell of plated bacteria suspensions (10^−1^ to 10^−8^ cfu/mL) was performed; also, identification of pure bacterial colonies was done using an API 20E bacterial identification kit. CLSI guidelines for antimicrobial susceptibility testing were adopted to determine the antibiotic susceptibility/resistance of the cultured bacterial isolates. Identification of resident‐resistant genes was done using molecular biology procedures. The results on viable cells in seepage samples ranged from 4.30 × 10^2^ to 1.29 × 10^9 ^cfu/mL. *Pseudomonas luteola, Enterococcus vulneris, Salmonella choleraesuis* spp *arizonae, Escherichia coli, Enterobacter cloacae, Proteus mirabillis* etc. were isolated from the pig farm soil samples. Almost all of the cultured isolates were resistant to Penicillin G, Vancomycin, Oxytetracycline, Spectinomycin, and Lincomycin. The most frequent resistant genes detected in the isolates were *Van A, Van B, InuA, aph* (*3”*)*‐llla, bla_TEM,_ Otr A,* and *Otr B*. It was inferred from the study that Pig farm seepage has the ability to cause bacterial pollution that may negatively impact the natural environment, by introducing bacteria pathogens that harbor antibiotic‐resistant genes.

## 1 INTRODUCTION

Pig farms are known to produce seepage with high concentration of pollutants. Recycling of this seepage in a sustainable manner remains a major challenge in agricultural sectors (Ramírez et al., [Link]). Mishandling of seepage results in the pollution of soil and water systems with nitrogen, phosphorus, bacteriological pathogens, and parasites, which in turn may impact negatively on the environment (Ramírez et al., [Link]). Surface run‐off of seepage from animal waste to the natural environment may negatively impact the health of plants, animals, and human beings. Applying seepage to land is an effective way of disposing of animal waste, and this solves the problem of removing animal waste and improves agricultural productivity, but the practice may distort the natural flora and fauna in the environments.

Antimicrobial resistance (AMR) has been raised as a global health concern, responsible for rising incidences of both enfeeble and lethal diseases (WHO, 2015). The continuous increase in resistance to established antibiotics by pathogens is a world crisis and has taken a center stage in prophylactic and curative medicine worldwide, most importantly in low‐income African countries (Ndihokubwayo et al., 2013). The problems likely to be caused by AMR acquisition in bacteria may be irreversible as this could result in limitations in disease pathology and therapeutic remedies (Ndihokubwayo et al., 2013). This will negatively impact on the environment and human health, most especially in developing countries such as South Africa, which lacks access to good‐quality medical treatments among the black majority, where bacterial infections are now becoming an important cause of morbidity and mortality (Samie et al., 2012).

Currently, the rapid increase in antibiotic drug resistance is more than the development of novel antibiotics drugs (Fahrenfeld, Ma, O'Brien, & Pruden, 2013). An increase in the incidences of AMR in bacteria may be due to mobile genetic elements that can be readily transferred through bacterial populations (Kumarasamy et al., 2010).

Unfortunately, disposing of seepage to agricultural land can as well introduce bacterial pollutants to the soil and groundwater in the surrounding environment (Obasi, Nwadinigwe, & Asegbeke, 2008). Mass storage of seepage may further be a serious hazard for biological balance in the environment (Muhibbu‐Din, Aduwo, & Adedeji, 2011). Bacteriological pollution of soil and water through agricultural practices usually has an overall effect on both animals and the natural environment (Toa, Ying, Su, Zhou, & Sidhu, 2010). Environmental pollution by bacterial pathogens may cause numerous diseases as a result of either ingestion or direct contact, or inhalation of contaminated aerosols (Tyrrel and Quiton, 2003).

Oxygen‐demanding substances, such as ammonia, nutrients (especially nitrogen and phosphorus), solids, pathogens, and odorous compounds, are the pollutants most commonly associated with seepage (Zhu et al., 2013). According to Madigan et al. (2000), the physical and chemical seepage treatment process has been developed to limit nitrogen and phosphorus pollution, but these treatment processes do not eliminate microbial pollution. Seepage discharge or spillage is a major component of water pollution that contributes to oxygen demand, nutrient loading, promotes toxicity, algal blooms that lead to the destabilization of the environment (González et al., 2009).

Fecal pathogens that are of environmental concern, and that may be detected in seepage, including *Escherichia coli* O157: H7, *Salmonella* spp., *Campylobacter* spp., *Shigella* spp., *Giardia lamblia*,* Cryptosporidium parvum,* and *Vibrio cholerae* (Obasi et al., 2008). Due to the extensive use of antibiotics in pig farms for disease control and as growth promoters (Sun et al., 2014), these bacteria can be overexposed to antibiotics and can hence develop a mechanism to resist the lethal effects of antibiotics. These bacteria, along with their antibiotic resistance genes, may be introduced to the environment through accidental spillage, surface run‐off, or overflow of pig farm seepage (Ghosh & Lapara, 2007).

Nonetheless, numerous studies on the multidrug resistance (MDR) profiling of bacteria have been focused more on isolates from clinical and food sources (Adefisoye & Okoh, 2016; Karczmarczyk, Abbott, Walsh, Leonard, & Fanning, 2011), with little researched information available on the MDR profiles of pathogenic bacteria pollution emanating from pig farms in South Africa. Agricultural wastewater effluents are considered hot spot or potential reservoirs for the dissemination of pathogens and antibiotic resistance genes in the environment due to the high use of antibiotics for disease treatment and growth promotion; hence, the need for such information becomes imperative. Therefore, the objectives of this study were to assess the level of bacteriological pollution emanating from the pig farm seepage and to identify the resident antibiotic‐resistant genes of prevailing bacteria.

## 2 METHODOLOGY

### 2.1 Study area and source of sampling

Water samples were collected monthly over a period of six months (March 2013 – August 2013) at the pig farm, ARC‐API. Water samples were collected in clean sterile glass bottles (cleaned with dilute Nitric acid (HNO_3_) and detergent, followed by distilled water). Samples were collected from four different sites on the pig farm, that is, pig enclosures (WW‐Enc), pig influent 2 m from the constructed wetland (Iff‐WW), pig farm constructed wetland for wastewater treatment (WW‐CW), and effluent 2 m from the constructed wetland (WW‐Eff). The ARC‐API is located 25 km south of Pretoria (25°52′S 28°13′E/25.867°S 28.217°E/‐25.867; 28.217) in Gauteng Province, Republic of South Africa.

### 2.2 Bacteria isolation

Wastewater samples (100 mL) were concentrated to 20 mL by centrifugation at 12,000 rpm for 10 min using Sorvall RC 26 plus (Labotech PTY [LTD]). Samples were analyzed by serial dilution method of tenfold up to 10^−8^ using sterile 0.9% (w/v) saline solution as a diluent (Bezuidenhout, Mthembu, Puckree, & Lin, 2002). The isolates were recovered using Nutrient agar, MacConkey agar, Xylose Lysine Deoxycholate agar (XLD agar), and Eosin Methylene Blue (EMB) agar, incubated at 37°C for 48 hr. Colonies on media plates were streaked on Nutrient agar to obtain pure isolates and subjected to preliminary identification using API 20E (bioMérieux South Africa (Pty) Ltd following manufacturer's guidelines). Pure isolates were streaked on Nutrient agar and incubated at 37°C for 24 hr. The overnight grown cultures were then inoculated into 5 mL of 0.85% (w/v) saline solution, and the turbidity of the resulting solution was adjusted to 0.5 McFarland Standard. The manufacture's procedure was followed in inoculating the isolates on the API 20E test strips. All reactions were read according to the calculated seven‐digit octal number, and the organism identity was determined using the apiweb.

### 2.3 Antibiotics resistance profiling

The antibiotic resistance/susceptibility profiling was determined by the Kirby‐Bauer disk diffusion method using the standard procedure of the Clinical and Laboratory Standards Institute (CLSI, 2011; Kumar, Tripathi, & Garg, 2013). What informed our choice of antibiotics is mainly to have representatives of antibiotic classes and generations, which are used as feed additive in pig production either as growth promoters and/or to manage and treat diseases and infections. The nineteen commercial antibiotic disks (Oxoid, UK) which include the following: Penicillin G (P)(10 µg), Sulphamethaxazole (RL) (25 µg), Vancomycin (VA) (30 µg), Ampicillin (AML) (10 µg), Amoxicillin (APR) (25 µg), Apramycin (AMP) (15 µg), Neomycin (N) (30 µg), Tilmicosin (TIL) (15 µg), Oxytetracyclin (OT) (30 µg), Spectinomycin (SH) (25 µg), Lincomycin (MY), (15 µg), Trimethoprim (TM) (2.5 µg), Nalidixic Acid (NA) (30 µg), Gentamicin (CAZ) (10 µg), Tetracycline (TE) (30 µg), Ceftazidime (CN) (10 µg), Norfloxacin (NOR) (10 µg), and Nitrofurantoin (NI) (300 µg) were employed for the susceptibility testing using Mueller Hinton agar (Oxoid, UK). The antibiotic resistance/susceptibility profile was determined by measuring zones of inhibition and comparing them to the Clinical and Laboratory Standards Institute (CLSI, 2011) interpretive chart. The experiments were performed in triplicates, and the average values were considered for patterns of antibiotic resistance or sensitivity. Multidrug resistance (multiple antibiotic resistance phenotypes) was determined as the exhibition of resistance to three or more different classes of antibiotics. The MDRI of each sample was estimated by the equation: MDRI = *a*/(*b* × *c*), where *a* represents the aggregate antibiotic resistance score of all isolates from the sample; *b* represents the number of antibiotics; and *c* represents the number of isolates from the sample (Krumperman, 1983).

### 2.4 Detection of the antibiotic resistance gene in identified isolates

#### 2.4.1 DNA Isolation

Bacterial DNA was isolated using NucleoSpin Tissue Genomic DNA purification kit (Machery‐Nagel). The manufacture's procedure was followed for isolation of the genomic DNA (support protocol for bacteria). The purity and yield of the DNA were assessed spectrophotometrically by calculating the A_260_/A_280_ ratios and the A_260_ values to determine protein impurities and DNA concentrations. The concentration and quality of the DNA were determined by agarose gel electrophoresis and spectrophotometer analysis (NanoDrop ND‐2000c, Thermo).

#### 2.4.2 PCR amplification assays for the detection of antibiotic resistance genes

Polymerase chain reaction with specific oligonucleotide primers was used to determine the presence/occurrence of antibiotic resistance genes (ARG) in isolates that showed multidrug resistance to the antibiotics tested. The detection of 26 ARGs targets cutting across different classes of antibiotics tested was analyzed following a previous protocol described by Hsu, Wang, Chen, Lu, & Chen (2007). Targeted antibiotic resistance genes were selected to represent those commonly reported for farm animals (especially pigs), animal products, and farm environment, and also on the probable abilities of these genes to be transferred to human pathogens after the consumption of meat and meat products. In this study, the ARGs screened are chosen from antibiotics, where microorganism had resistance of over 50%. The ARG targets include the following: (*aadA, aa(6’)‐le‐aph(2”)‐la, aph(2”)‐lb, aph(2”)‐lc, aph(2”)‐ld, aph(3”)‐llla, ant(4’)‐la, aac(3’)‐lv, VanA, VanB, VanC1, VanC2/C3, OtrA, OtrB, blaSHV, bla_TEM_, bla_OXA_, bla_VEB_, bla_PER_, Sul1, Sul2, Inu(A), Inu(B), Inu(C), Inu(D), Inu(F)*). The PCR products were analyzed by gel electrophoresis using 1.5% (w/v) agarose in 1X TBE buffer. The primers used for this study were previously validated, and the details of each ARG primer sequence and annealing temperature are described in Table 1. Amplifications of bacteria DNA were performed using iProof High Fidelity DNA Polymerase (BIO‐RAD) following manufactures guidelines but with amendments: the PCR mixture (20 µL) contained 0.02 U/µL iProof DNA Polymerase; 1X iProof HF Buffer; 3% DMSO; 700 µM MgCl_2_; 200 µM dNTPs; 0.5 µM Forward Primer; 0.5 µM Reverse Primer; 1 µg DNA template; and 11.4 µL of nuclease‐free water. The PCR assay conditions are shown in Table 2.

**Table 1:** Primer sequence and annealing temperature for detection of antibiotic resistance genes

Primers	Sequence (5' to 3')	Annealing Temperature	Type of resistance mediated	References
*aadA*	*F*‐5'TGATTTGCTGGTTACGGTCAG'3 R‐5'CGCTATGTTCTCTTGCTTTTG'3	53°C	Plasmid mediated (aminoglycoside resistance)	Vakulenko et al. (2003)
*aa(6*'*)‐le‐aph(2″)‐la*	*F*‐5'CAGGAATTTATCGAAAATGGTAGAAAAG'3 R‐5'CACAATCGACTAAAGAGTACCAATC'3	55°C		Vakulenko et al. (2003)
*aph(2″)‐lb*	*F*‐5'CTTGGACGCTGAGATATATGAGCAC'3 R‐5'GTTTGTACGCAATTCAGAAACACCCTT'3	58°C		Vakulenko et al. (2003)
*aph(2″)‐lc*	*F*‐5'CCACAATGATAATGACTCAGTTCCC'3 R‐5'CCACAGCTTCCGATAGCAAGAG'3	58°C		Vakulenko et al. (2003)
*aph(2″)‐ld*	*F*‐5'GTGGTTTTTACAGGAATGCCATC'3 R‐5'CCCTCTTCATACCAATCCATATAACC'3	56°C		Vakulenko et al. (2003)
*aph(3″)‐llla*	*F*‐5'GGCTAAAATGAGAATATCACCGG'3 R‐5'CTTTAAAAAATCATACAGCTCGCG'3	54°C		Vakulenko et al. (2003)
*ant(4*'*)‐la*	*F*‐5'CAAACTGCTAAATCGGTAGAAGCC'3 R‐5'GGAAAGTTGACCAGACATTACGAAACT'3	58°C		Vakulenko et al. (2003)
*aac(3*'*)‐lv*	*F*‐5'GTCGTCCAATACGAATGGCG'3 R‐5'CAGCAATCAGCGACCTTG'3	55°C		Vakulenko et al. (2003)
*VanA*	(F)CAT GAA TAG AAT AAA AGT TGC AAT A (R) CCC CTT TAA CGC TAA TAC GAT CAA	55°C	Chromosomal mediated (glycopeptide: vancomycin resistance)	Jánošková & Kmeť (2004)
*VanB*	(F)GTG ACA AAC CGG AGG CGA GGA (R)CCG CCA TCC TCC TGC AAA AAA	58°C		Jánošková & Kmeť (2004)
*VanC1*	(F)GGT ATC AAG GAA ACC TC (R)CTT CCG CCA TCA TAG CT	54°C		Jánošková & Kmeť (2004)
*VanC2/C3*	(F) CGG GGA AGA TGG CAG TAT (R) CGC AGG GAC GGT GAT TTT	55°C		Jánošková & Kmeť (2004)
*OtrA*	(F) GAACACGTACTGACCGAGAAG (R) CAGAAGTAGTTGTGCGTCCG	57°C	Ribosomal mediated (Oxytetracycline resistance)	Nikolakopoulou et al. (2005)
*OtrB*	(F) CCGACATCTACGGGCGCAAGC (R) GGTGATGACGGTCTGGGACAG	61°C	Efflux mediated (Oxytetracycline resistance)	Nikolakopoulou et al. (2005)
*bla_SHV_*	(F) ATGCGTTATATTCGCCTGTG (R) TTAGCGTTGCCAGTGCTCGA	53°C	Extended‐spectrum β‐lactamases resistance (Ceftazidime)	Jiang et al. (2006)
*bla_TEM_*	(F) ATGAGTATTCAACATTTTCG (R) TTACCAATGCTTAATCAGTG	47°C		Strateva et al. (2007)
*bla_OXA_*	(F) CGAGCGCCAGTGCATCAAC (R) CCGCATCAAATGCCATAAGTG	56°C		Strateva et al. (2007)
*bla_VEB_*	(F) CGACTTCCATTTCCCGATGC (R) GGACTCTGCAACAAATACGC	55°C		Strateva et al. (2007)
*bla_PER_*	(F) AATTTGGGCTTAGGGCAGAA (R) ATGAATGTCATTATAAAAGC	45°C		Strateva et al. (2007)
*Sul1*	*F*‐5' GGATCAGACGTCGTGGATGT'3 R‐5' GTCTAAGAGCGGCGCAATAC'3	62°C	Sulfonamide resistance	Faldynova et al. (2013)
*Sul2*	F'‐5' CGCAATGTGATCCATGATGT'3 R'‐5' GCGAAATCATCTGCCAAACT'3	60°C		Faldynova et al. (2013)
*Inu(A)*	(F) GGTGGCTGGGGGGTAGATGTATTAACTGG (R) GCTTCTTTTGAAATACATGGTATTTTTCGA	56°C	Chromosomal mediated (Lincomycin resistance)	Li et al. (2013)
*Inu(B)*	(F) CCTACCTATTGTTTGTGGAA (R) ATAACGTTACTCTCCTATTTC	50°C		Li et al. (2013)
*Inu(C)*	(F) AATTTGCAATAGATGCGGAGA (R) TCATGTGCATTTTCATCA	52°C		Li et al. (2013)
*Inu(D)*	(F) ACGGAGGGATCACATGGTAA (R) TCTCTCGCATAATAACCTTACGTC	55°C		Li et al. (2013)
*Inu(F)*	(F) CACCATGCTTCAGCAGAAAATGATC (R) TTACTTGTTGTGCGGCGTC	55°C		Li et al. (2013)

**Table 2:** Thermal cycling protocol for detection of ARG's

Cycle step	Temperature	Time	Number of cycles
Initial denaturing	98°C	30 s	1
Denaturing	98°C	10 s	35
Annealing	The annealing temperature of Primer (Table 1)	30 s	
Extension	72°C	30 s	
Final extension	72°C	10 min	1

All PCR experiments have positive control (*E. coli* ATCC 25922, *Ps. aeruginosa* ATCC 19429, *S. marscensce* ATCC 14041) and a blank control (reaction mixture with no DNA template). Amplified DNA from each sample (10 µL) was mixed with 1 µL of 6× loading buffer dye and loaded on a 1% horizontal agarose gel containing 0.5 mg/mL of ethidium bromide. A 100‐bp DNA ladder ranging from 100 to 3,000 bp (Thermo Scientific) was also added to each gel to confirm the size of amplified DNA bands. All gels were run in 1× TAE buffer at 5 V/cm for 30 min and visualized by UV trans‐illumination.

### 2.5 Data analysis

The general linearized model (GLM) of SAS was used to generate analysis of variance (ANOVA), means, standard error, range. The count of >106 CFU/mL indicates a contamination risk for animals and humans. All individual result recorded using Microsoft Excel 2010 software (Microsoft Corporation) and the bacteriological data were transformed in decimal logarithms. For antibiotic resistance gene: two‐way analysis of variances (ANOVA) were performed to test the significant difference in the antibiotic resistance frequency at different sampling sites, and critical *p*‐value was set at 0.05.

## 3 RESULTS

### 3.1 Results for bacteriological analysis

**Figure 1:**
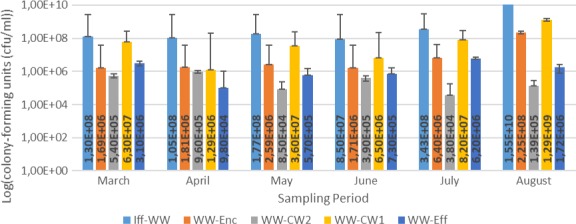
Results for bacteriological analyses of pig farm water samples on Nutrient agar. Key: WW‐Enc = enclosure water; Iff‐WW = influent 2 m away from constructed wetland; WW‐CW1 = constructed wetland 1; WW‐CW2 = construction wetland 2; WW‐Eff = effluent 2 m away from constructed wetland

**Figure 2:**
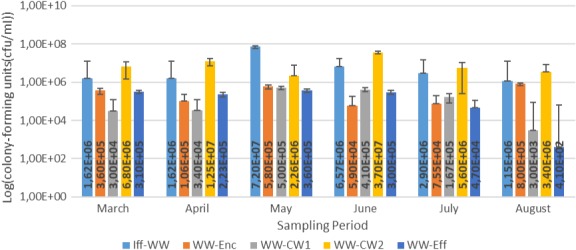
Results for Bacteriological analyses of pig farm water samples on EMB agar. Key: WW‐Enc = enclosure water; Iff‐WW = influent 2 m away from constructed wetland; WW‐CW1 = constructed wetland 1; WW‐CW2 = construction wetland 2; WW‐Eff = effluent 2 m away from constructed wetland

**Figure 3:**
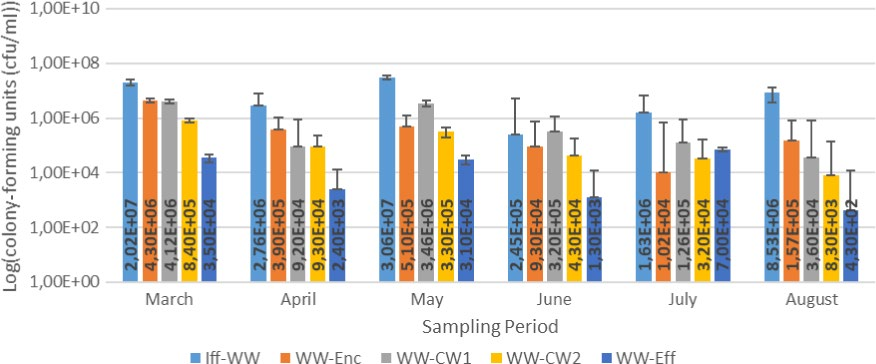
Results for Bacteriological analyses of pig farm water samples on XLD agar. Key: WW‐Enc = enclosure water; Iff‐WW = influent 2 m away from constructed wetland; WW‐CW1 = constructed wetland 1; WW‐CW2 = construction wetland 2; WW‐Eff = effluent 2 m away from constructed wetland

**Figure 4:**
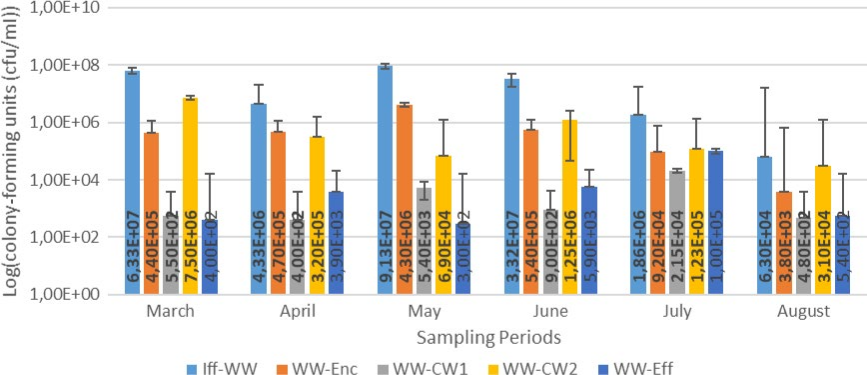
Results for bacteriological analyses of pig farm water samples on MacConkey agar. Key: WW‐Enc = enclosure water; Iff‐WW = influent 2 m away from the constructed wetland; WW‐CW1 = constructed wetland 1; WW‐CW2 = construction wetland 2; WW‐Eff = effluent 2 m away from the constructed wetland

Results for the bacterial enumeration of pig farm wastewater samples are shown in Figures 1, 2, 3, 4. In Nutrient agar (Figure 1), the colony‐forming cells ranged from 3.80 × 10^5^ cfu/mL to 1.29 × 10^9^ cfu/mL, and the results showed insignificant variation across sampling points and sampling months. In EMB agar (Figure 2), the colony‐forming cells ranged from 3.00 × 10^3^ cfu/mL to 7.20 × 10^7^ cfu/mL and the results varied significantly with regard to sampling points and months. The colony‐forming cells ranged from 4.30 × 10^2^ cfu/mL to 3.06 × 10^7^ cfu/mL in XLD agar (Figure 3), and the results did not vary significantly from sampling points but varied significantly (*p* < 0.1), monthly. In MacConkey agar, the colony‐forming cells ranged from 3.0 × 10^2^ cfu/mL to 9.13 × 10^7^ cfu/mL (Figure 4), the results varied insignificantly across sampling months and sampling points.

The results for the identification of 74 isolates from wastewater using API20E kit were determined by observing a change in color on the API20E test strip. A seven numeral code was used to identify the microorganism on apiweb software. The identified isolates were *Ps. Luteola, Escherichia vulneris, Salmonella choleraesuis spp arizonae, Escherichia coli 1, Enterobacter cloacae, Ps. Fluorescens/putida, Enterobacter aerogenes, Serratia ordoriferal, Pasteurella pneumotropica, Ochrobactrum antropi, Proteus vulgaris group, Proteus vulgaris, Salmonella* spp*, Aeromonas hydrophila/caviae/sobria1, Proteus mirabillis, Vibrio fluvials, Rahnella aquatillis, Ps. aeruginosa, Stenotrophomonas maltophilia, Klebsiela pneumoniae, Cedecea davisae, Serratia liquefaciens, Serratia plymuthica, Enterobacter sakaziki, Citrobacter braakii, Enterobacter amnigenus 2, Yersinia pestis, Serratia ficaria, Enterobacter gergoriae, Enterobacter amnigenus 1, Serratia marcescens, Raoutella terrigena, Hafnia alvei 1, Providencia rettgeri, and Pantoa*.

**Figure 5:**
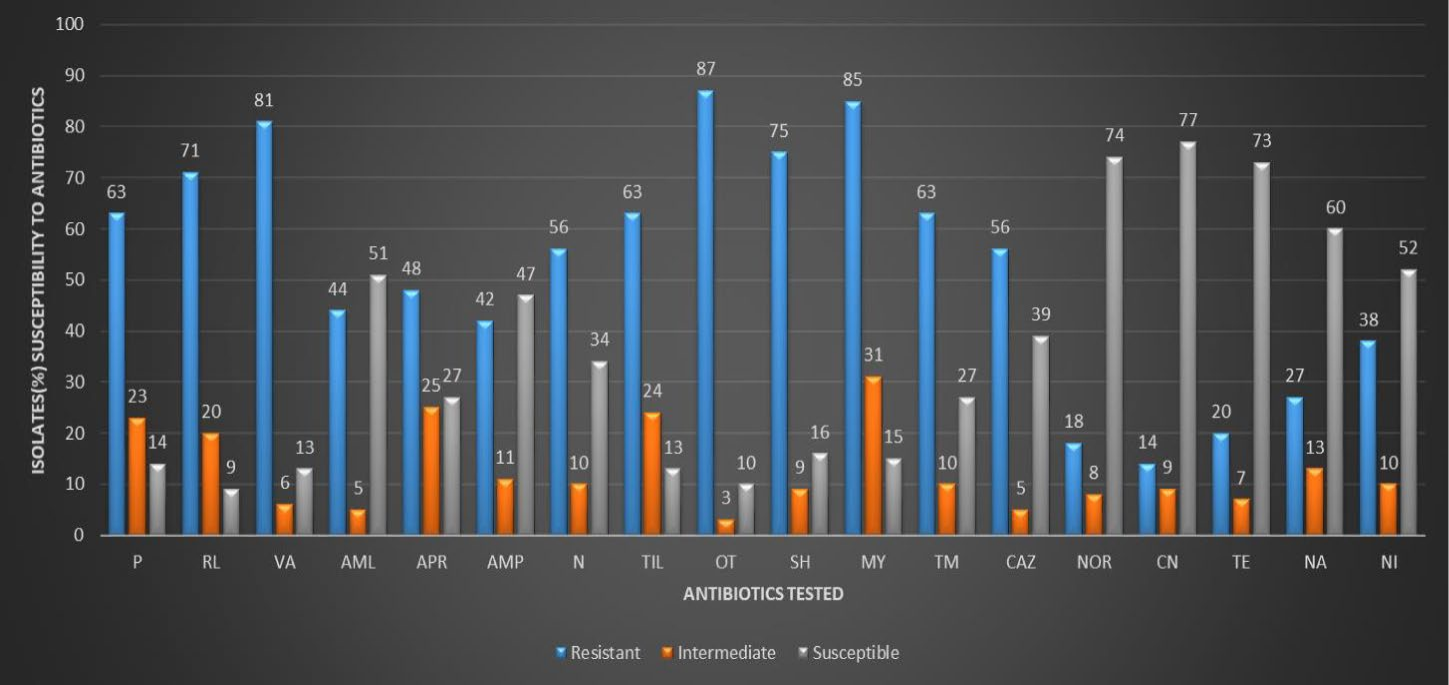
Results of susceptibility analyses of 18 different antibiotics used to test antibiotic sensitivity in isolates. Penicillin G (P), Sulphamethaxazole (RL), Vancomycin (VA), Ampicillin (AML), Amoxicillin (APR), Apramycin (AMP), Neomycin (N), Tilmicosin (TIL), Oxytetracycline (OT), Spectinomycin (SH), Lincomycin (MY), Trimethoprim (TM). Nitrofurantoin (NI), Nalidixic Acid (NA), Norfloxacin (NOR), Oxytetracycline (OT), Tetracycline (TE), Gentamicin (CAZ), Ceftazidime (CN)

The results of susceptibility analysis using 19 different antibiotics are shown in Figure 5. The Figure shows the resistance (R), susceptibility (S), and intermediate (I) levels of isolates to tested antibiotics. The results (Figure 5) showed that isolates were resistant to Penicillin G, (63%), Sulphamethaxazole (71%), Spectinomycin (71%), Tilmocosin (63%), Lincomycin (79%), and Trimothoprim (63%), Neomycin (56%), and Gentamycin (56%). The highest resistance to screened antibiotics was observed on Oxytetracycline (87%), Lincomycin (85%), and Vancomycin (81%). Of all screened antibiotics, a large proportion of isolates were susceptible to Norfloxacin (74%), Ceftadizime (77%), Tetracycline (73%), Nalidixic acid (60%), and Nitrofurantoin (52%). With respect to Ampicillin and Apramycin, the percentage of susceptible isolates (51% and 47%, respectively) compared to those that were resistant (44% and 42%, respectively) were more or less the same.

The results for Multiple Antibiotic‐ resistance Phenotypic (MARP) and Multidrug Resistance Index (MRDI) are reported in Table 3. The most observed MARP patterns observed were P‐RL‐VA‐AML‐APR‐AMP‐N‐TIL‐OT‐SH‐MY‐TM in 15 isolates, and P‐RL‐VA‐APR‐N‐TIL‐OT‐SH‐MY in six isolates. The MDRI was estimated to range from 0.2 to 1 in all the isolates tested. Among the 74 phenotype patterns, the most observed were P‐RL‐VA‐AML‐APR‐AMP‐N‐TIL‐OT‐SH‐MY‐TM at a frequency of 78.95%, and P‐RL‐VA‐APR‐N‐TIL‐OT‐SH‐MY at a frequency of 31.60% in six isolates. Some of the resistance patterns were not frequently detected, and the isolates were found to be resistant to only one antimicrobial agent. Multidrug Resistance Index was also observed to be high with 10 isolates having an MDRI of 100%, and 12 isolates had MDRI ranging from 75% to 92%. The MDRI ranged from 25% to 100% with the mean of 78.14%.

**Table 3:** The predominant multiple antibiotic resistance phenotypes and multidrug‐resistant Index of isolates

Multiple antibiotic resistance phenotype (MARP)			Multidrug‐resistant index (MDRI)			
Phenotype	Number(s) of Isolates	Percentage (%)	Isolates	MDRI (%)	Isolates	MDRI (%)
VA‐SH‐TM	2	10.50	EFF4a	100	CW1–3	83
SH‐MY‐TM	2	10.50	EFF6	100	IFF4	25
RL‐APR‐TIL‐SH‐MY‐TM	2	10.50	EW8	100	IFF5	25
P‐VA‐TIL‐OT‐SH‐MY	2	10.50	EW1	100	IFF6	83
P‐RL‐VA‐TIL‐OT‐SH‐MY	2	10.50	EW1	75	IFF7	25
P‐RL‐VA‐APR‐TIL‐OT‐MY	2	10.50	EW10	58	IFF9	92
P‐RL‐VA‐APR‐N‐TIL‐OT‐SH‐MY	6	31.60	EFF3	100	IFF8	58
P‐RL‐VA‐APR‐AMP‐N‐TIL‐OT‐SH‐MY‐TM	3	17.80	EW14	75	IFF1	75
P‐RL‐VA‐AML‐APR‐AMP‐TIL‐OT‐SH‐MY‐TM	3	17.80	EW11	75	IFF20	83
P‐RL‐VA‐AML‐APR‐AMP‐TIL‐OT‐SH‐MY	4	21.05	EW12	67	IFF3	42
P‐RL‐VA‐AML‐APR‐AMP‐OT‐SH‐MY‐TM	2	10.50	EW9	75		
P‐RL‐VA‐AML‐APR‐AMP‐N‐TIL‐OT‐SH‐MY‐TM	15	78.95	EW7	100		
P‐RL‐VA‐AML‐APR‐AMP‐N‐TIL‐OT‐SH‐MY	4	21.05	EFF2	100		
P‐RL‐VA‐AML‐AMP‐TIL‐OT‐SH‐MY‐TM	2	10.50	EFF5	100		
P‐RL‐VA‐AML‐AMP‐SH‐MY‐TM	2	10.5	EFF15	100		
P‐RL‐VA‐AML‐AMP‐N‐TIL‐OT‐SH‐MY	2	10.50	EFF1	92		
P‐AML‐AMP‐OT‐SH‐TM	2	10.50	EW3	75		
OT	2	10.50	EFF4	100		
MY	2	10.50	EW2	83		

Note

The table shows the most occurring phenotype antibiotic‐resistant patterns and shows isolates with the highest MDIR where 10 isolates showed 100% MDRI. Isolate had up to 19 phenotypes multiple resistance. Most isolates had predominant P‐RL‐VA‐AML‐APR‐AMP‐N‐TIL‐OT‐SH‐MY‐TM (78.95%), and P‐RL‐VA‐APR‐N‐TIL‐OT‐SH‐MY (31.60%) phenotype multiple resistance. About 55 isolates had more than five phenotype antibiotic resistance patterns where Penicillin G (P), Sulphamethaxazole (RL), Vancomycin (VA), Ampicillin (AML), Tilmicosin (TIL), Oxytetracycline (OT), Spectinomycin (SH), Lincomycin (MY) were the most predominant.

Results for the detection of resistance gene are shown in Tables 4 and 5. The results show that most isolates possess *aa (6’)‐le‐aph (2”)‐la* gene, *aph (2”)‐lb* gene, *aph (3”)‐llla* genes for aminoglycosides resistance, *Sul1* gene and *Sul2* gene for Sulphamethaxazole resistance, *VanA*,* VanB,* and *VanC2/C3* resistance genes for vancomycin, *Inu A* and *Inu C* resistance genes for lincomycin, *OtrA* and *OtrB* resistance genes for oxytetracyclines and bla_TEM_ and bla_PE_ resistance gene for beta‐lactamase resistance. Only three isolates, namely *E. vulneris*,* Salmonella* spp, and *Cedecea davisae,* were observed to have *aadA* resistance gene. *E. vulneris*,* Enterobacter cloacae*,* Ochrobactrum antropi*,* Ochrobactrum antropi*,* Enterobacter gergoriae*,* Enterobacter amnigenus 1*,* Pantoa* had *aph (2”)‐lc* resistance gene. Eleven isolates had *aac (3’)‐lv*, four isolates had *VanC* and *InuB*, seven isolates had InuF, six isolates had bla_SHV_, and eight isolates had bla_OXA_. Only *E. coli* had *aph (2”)‐ld* resistance gene, and only a *Salmonella choleraesuis* spp *arizonae* had *VanD* resistance gene. *Proteus mirabilis* and *Enterobacter amnigenus* were the only isolates that had *InuD* gene.

**Table 4:** Results for detection of resistance genes in isolate

Isolates	Antibiotic resistance genes																										
	*aadA*	*aa(6*'*)‐le‐aph(2″)‐la*	*aph(2″)‐lb*	*aph(2″)‐lc*	*aph(2″)‐ld*	*aph(3″)‐llla*	*ant(4*'*)‐la*	*VanA*	*VanB*	*VanC*	*VanC2/C3*	*VanD*	*InuA*	*InuB*	*InuC*	*InuD*	*InuF*	*bla_TEM_*	*bla_SHV_*	*bla_OXA_*	*bla_VEB_*	*bla_PER_*	*OtrA*	*OtrB*	*aac(3*'*)‐lv*	*Sul1*	*Sul2*
*Ps. luteola*	−	−	−	−	−	−	−	+	+	−	+	−	−	−	+	−	−	+	−	−	−	+	−	+	−	−	−
*E. vulneris*	+	+	+	+	−	−	−	+	+	−	−	−	+	‐	−	−	−	+	−	+	−	+	+	+	−	−	−
*Salmonella choleraesuis* spp *arizonae*	−	+	−	−	−	+	−	+	+	−	+	+	−	−	−	−	−	−	−	+	−	−	+	+	+	+	+
*E. coli 1*	−	−	−	−	+	−	−	+	+	−	−	−	−	−	−	−	−	−	+	+	−	+	+	+	−	−	+
*Enterobacter cloacae*	−	−	+	+	−	+	−	+	+	+	+	−	+	−	−	−	−	−	−	+	−	−	+	+	+	−	−
*Ps. flourescens/putida*	−	−	−	−	−	−	−	+	+	−	+	−	−	−	−	−	−	+	−	−	−	+	+	+	−	−	−
*Enterobacter aerogenes*	−	−	+	−	−	+	−	+	+	−	+	−	+	−	+	−	−	+	−	−	−	−	+	+	+	+	+
*Serratia ordoriferal*	−	−	+	−	−	−	−	−	+	−	+	−	−	−	−	−	−	−	+	−	−	+	+	+	−	−	+
*Pasteurella pneumotropica*	−	+	−	−	−	−	−	+	+	−	+	−	+	−	+	−	−	+	−	+	−	−	+	−	−	−	+
*Ochrobactrum antropi*	−	+	+	+	−	+	−	−	−	−	−	−	+	−	+	−	−	−	−	−	−	+	+	+	−	+	−
*Proteus vulgaris group*	−	−	−	−	−	+	−	−	+	−	+	−	−	−	−	−	−	−	−	+	−	−	+	−	−	−	+
*Proteus vulgaris*	−	+	−	−	−	+	−	+	+	−	+	−	+	−	+	−	+	−	−	−	−	+	−	+	+	−	+
*Salmonella* spp	+	−	−	−	−	+	−	+	+	−	−	−	+	−	−	−	+	+	−	−	−	−	+	+	−	−	−
*Aeromonas hydrophila/caviae/sobria1*	−	+	+	−	−	+	−	+	+	−	+	−	+	−	+	−	+	+	+	−	−	−	+	+	−	+	+
*Proteus mirabillis*	−	−	−	−	−	+	−	+	+	+	−	−	−	−	+	+	−	+	−	−	−	+	+	+	−	−	+
*Vibrio fluvials*	−	+	−	−	−	+	−	+	+	−	+	−	+	−	−	−	−	−	−	−	−	−	+	+	−	−	−
*Rahnella aquatillis*	−	−	+	−	−	+	−	−	−	−	−	−	−	−	−	−	−	−	−	−	−	−	−	−	−	−	−
	17	17	17	17	17	17	17	17	17	17	17	17	17	17	17	17	17	17	17	17	17	17	17	17	17	17	17
Total number of isolate possessing tested ARG	2	7	7	3	1	11	0	13	15	2	11	1	9	0	7	1	3	8	3	6	0	8	14	14	4	4	9

Note

+: Antibiotic resistant gene detected; −: no antibiotic resistance gene detected; ARG: antibiotic resistance gene.

**Table 5:** Results for detection of resistance genes in isolate (Continue)

Isolates	Antibiotic resistance genes																										
	*aadA*	*aa(6*'*)‐le‐aph(2″)‐la*	*aph(2″)‐lb*	*aph(2″)‐lc*	*aph(2″)‐ld*	*aph(3″)‐llla*	*ant(4*'*)‐la*	*VanA*	*VanB*	*VanC*	*VanC2/C3*	*VanD*	*InuA*	*InuB*	*InuC*	*InuD*	*InuF*	*bla_TEM_*	*bla_SHV_*	*bla_OXA_*	*bla_VEB_*	*bla_PER_*	*OtrA*	*OtrB*	*aac(3*'*)‐lv*	*Sul1*	*Sul2*
*Ps. aeruginosa*	−	−	−	−	−	−	−	+	+	−	+	−	+	−	+	−	−	−	−	+	−	+	+	+	−	−	+
*St. maltophilia*	−	+	−	−	−	+	−	−	−	−	−	−	−	−	+	−	+	+	−	−	−	−	−	−	−	+	+
*Klebsiela pneumoniae*	−	−	−	−	−	−	−	+	+	−	+	−	+	−	−	−	−	+	−	−	−	−	+	−	−	−	+
*Cedecea davisa*	+	+	+	−	−	+	−	−	−	−	−	−	+	−	+	−		+	−	−	−	−	−	−	−	−	+
*Serratia liquefaciens*	−	−	+	−	−	+	−	−	+	+	−	−	+	−	−	−	+	+	−	−	−	−	+	−	−	+	−
*Serratia plymuthica*	−	−	+	−	−	+	−	−	−	−	−	−	+	+	−	−	−	−	−	−	−	−	+	+	+	−	−
*Enterobacter sakaziki*	−	−	+	−	−	+	−	+	+	−	−	−	+	−	−	−	−	+	+	−	−	−	+	+	−	+	+
*Citrobacter braakii*	−	+	−	−	−	+	−	−	+	−	+	−	−	−	+	−	−	−	−	−	−	−	−	−	+	+	−
*Enterobacter amnigenus 2*	−	−	−	−	−	+	−	+	+	−	−	−	−	+	+	−	+	+	−	−	−	+	+	+	−	+	−
*Yersinia pestis*	−	−	−	−	−	+	−	+	+	+	+	−	+	−	−	−	−	−	+	−	−	−	+	+	−	+	+
*Serratia ficaria*	−	+	+	−	−	+	−	+	+	−	−	−	−	+	+	−	−	+	+	−	−	−	+	+	+	+	+
*Enterobacter gergoriae*	−	−	+	+	−	−	−	+	+	−	+	−	−	−	−	−	−	−	−	−	−	+	+	+	+	−	−
*Enterobacter amnigenus 1*	−	+	−	+	−	−	−	+	+	−	+	−	−	−	−	+	+	+	−	−	−	+	+	+	−	−	−
*Serratia marcescens*	−	−	−	−	−	+	−	+	+	−	−	−	+	−	−	−	−	+	−	−	−	−	+	+	+	−	+
*Raoutella terrigena*	−	−	−	−	−	+	−	+	+	−	+	−	+	+	−	−	−	+	−	−	−	−	+	−	−	+	−
*Hafnia alvei 1*	−	−	−	−	−	+	−	−	−	−	−	−	−	−	−	−	−	−	−	−	−	−	−	−	−	+	−
*Providencia rettgeri*	−	+	+	−	−	+	−	−	−	−	−	−	−	−	+	−	−	−	−	+	−	−	+	−	−	−	−
*Pantoa*	−	+	+	+	−	+	−	−	−	−	−	−	+	−	+	−	−	−	−	−	−	+	−	+	+	−	−
Number of isolates tested	18	18	18	18	18	18	18	18	18	18	18	18	18	18	18	18	18	18	18	18	18	18	18	18	18	18	18
Total number of isolate possessing tested ARG	1	7	8	3	0	14	0	10	12	2	7	0	10	4	8	1	4	10	2	1	0	5	13	10	6	9	8

Note

+: Antibiotic resistant gene detected; −: no antibiotic resistance gene detected; ARG: antibiotic resistance gene.

## 4 DISCUSSION

The results for bacterial densities (Figures 1, 2, 3, 4) were observed to be high in enclosures and influent as compared to other sampling points. These densities were higher in all sampling points in this study and were higher than those recommended by DWAF and Government Gazette, where viable cells are recommended not to exceed 1,000 cfu/mL (DWAF, 1996c). The seepage from pig farm should be further treated, that is, chemical treatment by chlorination or by UV treatment should be applied to reduce the bacterial load. The biological evolution of soil and water habitats may be compromised if the seepage finds its way to the environment (Vaz‐Moreira, Nunes, & Manaia, 2014). Seepage inadequately debugged may introduce resistant bacteria and ARGs into the receiving environments, thus causing mobile genetic elements carrying ARGs or naked ARGs to be transferred to indigenous bacteria or other habitats. Sasáková et al. (2007) reported viable cell counts in a range of 9.8 × 10^6^ to 9.2 × 10^8^ cfu/mL from a pig farm seepage bacterial analysis; a range count, similar to what we have reported in this study. On the other hand, the results in this study were higher than those observed by Tymczyna, Chmielowiec‐Korzeniowska, & Saba (2000), where bacterial densities from environmental samples in the vicinity of pig farm ranged from 1.00 × 10^4^ to 3.00 × 10^4^ cfu/mL.

Detection of bacterial pathogens in the seepage may be attributed to the high load of animal excreta and serves as a pointer for possible bacteriological pollution that may have an effect on the soil ecological balance and aquatic life (Ezeronye and Ubalua, 2005). The detection of *Escherichia* spp., *Salmonella* spp., *Proteus* spp., *Pseudomonas* spp., *Klebsiella* spp., and *Enterobacter* spp. in pig farm seepage is of great concern; these bacteria are reported to be threats to the public health and food insecurity (Jandhyala et al., 2015). If the seepage can reach water systems, the bacteria might initiate various waterborne diseases (Jandhyala et al., 2015), such as diarrhea, urinary tract infections, respiratory infections, septic arthritis, fever, and vomiting in humans, and in severe cases may lead to death (Humphries & Linscott, 2015).

This study revealed the presence of AMR in *Pseudomonas* spp., against all primary antibiotics tested (Penicillin G, Ceftazidime, Gentamicin etc.). Penicillin G, Ceftadizime, and Gentamicin are the primary defense antibiotics used in treating *Pseudomonas* infections in humans (Humphries & Linscott, 2015). Also, the detection of *Yersinia pestis* with Sul1 and Sul2 resistance genes in the studied pig farm wastewater is of great concern. *Yersinia* spp. are reported to be extremely virulent pathogens that are likely to cause severe illnesses and plague infections in human which may lead to death (Duan et al., 2014). Sul1 and Sul2 are genes responsible for trimethoprim–sulfamethoxazole resistance, antibiotics considered to be the first line of drugs in treating bubonic plaque in humans, also, sulfonamides, in combination with trimethoprim, are for the treatment of diarrhea in weaned pigs (De Briyne et al., 2014). In a study by Dubinský et al. (2000), the authors identified and detected *Salmonella* spp*., E. coli, Yersinia* spp. in pig farm seepage. The results obtained in this study were similar to those observed by Tymczyna et al., 2000, in that study, bacteria such as *Salmonella* spp., *Klebsiella* spp.*, Pseudomonas* spp.*, Proteus* spp.*, Enterobacter aerogenes, and Citrobacter* spp. were isolated from the environmental water samples near the pig farm.

Results observed during the antibiotic resistance profiling (Figure 5) indicate that these organisms are well exposed to antibiotics at the pig farm and have developed mechanisms to evade or avoid the effects of these tested antibiotics. The most probable route of encounter of these isolates with antibiotics will be through the feed, water, and antibiotics used as prophylaxis; the farm where the samples are collected relies heavily on the use of antibiotics for growth promotion and for the management of diseases. The detection of antimicrobial resistance in bacterial pathogens is of great concern because most antibiotics used for animal production are similar to those used in humans (De Briyne et al., 2014). A possible explanation for the resistance to several antibiotics tested in this study could be the acquisition of a multidrug resistance plasmid and acquisition of a single mobile genetic cassette harboring genes coding for several different resistance mechanisms (von Wintersdorff et al., 2016). When this transfer of mobile genetic element between bacteria occurs, the antibiotic resistance could support their environmental dissemination independent of their original host (Heuer, Schmitt, & Smalla, 2011). Apart from the factors mentioned above, other factors such as disinfectants and heavy metals used in the pig farm may also have contributed to the maintenance of antibiotic resistance in bacteria (Schluter, Szczepanowski, Puhler, & Top, 2007). Results in this study were similar to those obtained by Dubinský *et al*. (2000), Kainer et al. (2007), and Werner et al. (2008); these authors observed resistance to penicillins, lincosamides, vancomycin, and an aminoglycoside, in the bacterial isolates reported.

Copious presence of bacteria with AMR genes in the samples from the vicinity of pig farm indicates that there is no proper treatment of the pig farm wastewater. The presence and abundance of Enterobacteriaceae in the water samples in pig farm not only revealed resistance to antibiotics commonly used in their treatment; it revealed the abundance of multidrug resistance genes in prevailing bacteria. If bacteria with such resistance to a broad spectrum of antibiotics across antibiotic classes become waterborne or airborne, farm workers and residents close to the pig farm are at high risk of infections.

The multiple antibiotic resistance phenotypes (Table 1) observed in this study showed that the isolates were resistant to more than three antibiotics, where 15 isolates were observed to be resistant to all antibiotics tested. The multidrug resistance index (MDRI) of isolates was also observed to be high (Table 1). The MDRI has been used to estimate the health risk associated with the spread of drug resistance in the environment. The MDRI of about 0.2 (arbitrary) is commonly used to differentiate between low health risk and high health risk. Thus, MDRI greater the 0.2 in the isolates suggest that the bacteria are from an environment of highly contaminated or high use of antibiotics (Adefisoye & Okoh, 2016). The MDRI observed in this study ranged between 0.2 and 1 suggests that the environment in the vicinity of pig farm is contaminated with bacteria containing antibiotic resistance genes.

The high MDRI values obtained in this study could be as a result of the exposure of the isolates to antibiotics pressure. This could have resulted from the inappropriate use of antibiotics in pigs for growth promotion or for treating diseases. This may be attributed probably, to the transfer of resistance genes between pathogens emanating from pig farm seepage and indigenous soil and aquatic microorganisms. Furthermore, the resistance gene transfer between non‐pathogenic and pathogenic bacteria may be a factor of both internal and external influences to the bacterium. External influences are factors that assist to facilitate DNA transferability such as temperature, pH, detergents, and organic solvents (Jury, Vancov, Stuetz, & Khan, 2010), while internal influences include factors such as the “SOS” response to DNA damage, which may result in increased frequency of transfer of certain resistance traits. This SOS response may regulate transcription when reacting to external stresses such as UV radiation and certain antibiotics (ciprofloxacin, trimethoprim, and β‐lactams), and may cause metabolic changes and mutations facilitating survival and resistance in bacteria, in the natural environment (Cirz *et al*., 2007). Mismanagement of antibiotics may lead to further development of multidrug resistance overtime if appropriate measures are not taken.

Kotzamanidis et al., 2009 observed that AML‐CAZ‐VA‐TE was the most occurring phenotype pattern in isolates from pig farm environment, but in this study, P‐RL‐VA‐AML was the most occurring phenotype observed, on the other hand, phenotype patterns observed in this study were similar to those observed by Kainer et al. (2007) and Werner et al. (2008). One major implication of multiple antibiotic resistance in pathogens is the limited treatment options for some bacterial infections that were previously thought to be curable. This could have huge public health implications (Adefisoye & Okoh, 2016).

Zhu et al. (2013) reported *Sul* resistance genes as the most frequently detected ARGs in pig farm seepage; similar results were our observations in this study (Table 4). Although, the results for *Sul* resistance gene detection in this study were observed to be lower than those reported by McKinney et al. (2010) where a high abundance of sulfonamide (*Sul1 and Sul2*) resistance genes in pig farm seepage was reported. Furthermore, results for resistance gene detection, as shown in this study, were also consistent to those observed by Munir & Xagoraraki (2011). The abundance of *Inu F* resistance gene in this study was lower than those observed by Cheng et al. (2013); Li et al. (2013). Detection of *aph (3’)‐IIIa* and *bla_TEM_* in this study were similar to those observed by Sun et al. (2014). Other AMR genes observed in this study include the following: *VanA, VanB*, InuA, *aph (3”)‐llla*, blaTEM, OtrA, and *OtrB* were observed to be the most detected resistant genes in this study.

## 5 CONCLUSION

As observed in this study, the bacterial colony‐forming units in the studied pig farm seepage were higher than the recommended limits (DWAF, 1996c), also, pathogens with multiple antibiotic resistance genes were detected. These are indicators of public health risks; therefore, it is inferred that pig farm seepage may contribute to bacterial pollution, which could burden the flora and fauna of the adjoining natural environment within the vicinity of the studied pig farm, by introducing bacterial pathogens that are carriers of multiple antibiotic resistance genes.

It is therefore suggested that more effort should be focused on the ARGs elimination from agricultural wastewater before the release to the environment, rather than focus on mitigation efforts after improper discharge into the environment. Further studies are needed to connect the diversity and variation in ARGs and the host bacteria and to shed light on the resistome of both pristine and anthropogenic impacted environments.

## CONFLICT OF INTEREST

Authors declare that there are no conflict of interests.

## AUTHORS CONTRIBUTION

Matjuda D.S. was the project coordinator and compiling of manuscript. Aiyegoro O.A. was the project manager, internal reviewer, and compiling of manuscript.

## ETHICS STATEMENT

Ethical clearance was not applicable to this study as no animals were used.

## Data Availability

All data are provided in full in the results section of this paper.
